# A retrospective study on the associations of serum 25-hydroxyvitamin D with N-terminal pro-B-type natriuretic peptide and left ventricular ejection fraction in patients with type 2 diabetes mellitus

**DOI:** 10.3389/fendo.2026.1943266

**Published:** 2026-10-28

**Authors:** Yuting Shao, Shasha Shi, Yifei Li, Guangyi Gao, Xuelin Feng, Minghui Li, Yuantao Liu, Yonggang Song

**Affiliations:** 1 Department of Endocrinology, The People’s Hospital of Jimo, Qingdao, China; 2 Department of Clinical Laboratory, The People’s Hospital of Jimo, Qingdao, China; 3 Department of Clinical Nutrition, Qilu Hospital (Qingdao), Cheeloo College of Medicine, Shandong University, Qingdao, China; 4 Department of Endocrinology, Qilu Hospital (Qingdao), Cheeloo College of Medicine, Shandong University, Qingdao, China

**Keywords:** 25-hydroxyvitamin D, left ventricular ejection fraction, N-terminal Pro-B-type natriuretic peptide, type 2 diabetes mellitus, urinary microalbumin-to-creatinine ratio

## Abstract

**Background:**

Accumulating evidence points to a role of vitamin D deficiency in cardiovascular diseases. However, its implications for cardiac dysfunction, particularly in patients with type 2 diabetes mellitus (T2DM), have not been well defined. In the present study, the relationships between serum 25-hydroxyvitamin D (25(OH)D) and markers for cardiac dysfunction were investigated.

**Methods:**

This retrospective study enrolled 601 participants (210 males, 391 females) with T2DM, aged ≥18 years. Patients with a history of vitamin D supplementation, hyper-/hypoparathyroidism, or incomplete data were excluded. Correlation analyses were conducted to explore the associations of N-terminal pro-B-type natriuretic peptide (NT-proBNP) and left ventricular ejection fraction (LVEF) with serum 25(OH)D and related clinical indexes. Multivariate logistic regression analysis was performed to explore the association between vitamin D deficiency and elevated NT-proBNP. Causal mediation analyses were performed to examine the potential mediating role of urinary microalbumin-to-creatinine ratio (UACR) in the association between vitamin D deficiency and NT-proBNP. A directed acyclic graph (DAG) was constructed to identify confounders for multivariable adjustment and distinguish mediators.

**Results:**

Of all subjects, 143 (24%) presented vitamin D deficiency and 221 (36.8%) presented vitamin D insufficiency. Elevated NT-proBNP was detected in 266 (44.3%) patients. In patients with vitamin D deficiency, NT-proBNP level was significantly increased compared with those with vitamin D sufficiency or those with vitamin D insufficiency, but LVEF showed no differences among groups. Multivariate logistic regression analysis showed that the odds ratio (OR) for elevated NT-proBNP in patients with vitamin D deficiency was 2.11 (95% CI 1.38-3.2, *P* < 0.001) versus patients with vitamin D sufficiency. After adjusting for age, sex, BMI, diabetes duration, hypertension, coronary heart disease, HbA1c, and eGFR, the association between vitamin D deficiency and elevated NT-proBNP remained significant (OR 1.833, 95% CI 1.115-3.013, *P* = 0.017). Mediation analysis revealed that approximately 43.23% of the total effect was statistically compatible with an indirect pathway through UACR.

**Conclusions:**

Vitamin D deficiency was associated with elevated NT-proBNP, a marker of myocardial wall stress that may signal early cardiac involvement, whereas no association with LVEF was observed. This association was partially accounted for by UACR in exploratory mediation analyses.

## Introduction

Vitamin D is an indispensable fat-soluble steroid hormone classically recognized for its role in calcium homeostasis and bone metabolism (1). Beyond these canonical effects, accumulating clinical evidence has further established its critical protective roles in the cardiovascular system. Vitamin D exerts cardioprotective effects through anti-inflammatory, antifibrotic, and antihypertrophic effects (2–4). In addition, vitamin D plays a critical role in maintaining vascular endothelial integrity by upregulating endothelial nitric oxide synthase (eNOS) expression, enhancing nitric oxide bioavailability, and suppressing endothelial inflammatory responses, and thus participates in the regulation of cardiac and renal microcirculation (5, 6). These vascular protective effects are particularly relevant in metabolic diseases such as type 2 diabetes mellitus (T2DM).

In recent years, the prevalence of T2DM has been rising rapidly as a pandemic worldwide. T2DM was shown to be associated with multiple cardiac complications, including myocardial infarction (MI), ventricular remodeling, and heart failure, etc (7, 8). Concurrently, low circulating vitamin D is highly prevalent among T2DM patients. A 2023 meta-analysis of 54 studies (n=38,016) reported that nearly 64% of T2DM patients were vitamin D deficient (9). The interplay between vitamin D deficiency and diabetic cardio-renal disease is increasingly recognized (10, 11). In T2DM, chronic hyperglycemia induces systemic microvascular endothelial dysfunction, which simultaneously compromises the glomerular filtration barrier—manifested as elevated UACR—and impairs coronary microvascular perfusion, ultimately increasing cardiac wall stress (12, 13). Vitamin D may modulate this axis through multiple convergent pathways: by preserving glomerular endothelial integrity via the VDR-eNOS axis, suppressing renal renin-angiotensin-aldosterone system (RAAS) activation, and attenuating low-grade inflammation that drives both albuminuria and myocardial strain (5, 14–16). Consequently, vitamin D deficiency may represent a common upstream perturbation that simultaneously predisposes to renal microvascular injury (elevated UACR) and subclinical myocardial strain in T2DM patients (17, 18).

UACR occupies a unique position at the intersection of renal and cardiovascular pathology in T2DM (19). As the earliest detectable marker of diabetic nephropathy, it reflects glomerular endothelial injury; yet it also serves as a surrogate of systemic microvascular dysfunction, since identical pathological processes concurrently damage glomerular and coronary microcirculation (20, 21). This dual nature underpins its hypothesized role as a mediator. We posit that vitamin D deficiency promotes glomerular endothelial injury and subsequent UACR elevation. Elevated UACR in turn reflects systemic microvascular damage and is accompanied by volume overload, both of which increase ventricular wall strain and promote myocardial strain. Accordingly, we hypothesized that UACR acts as a mediator along the hypothesized pathway from vitamin D deficiency to myocardial strain, and constructed a DAG to illustrate this framework (**Supplementary Figure S1**).

NT-proBNP is a stable peptide fragment derived from the proteolytic cleavage of pro-B-type natriuretic peptide. It is predominantly produced and released by ventricular myocardial cells when the heart is exposed to mechanical stress including myocardial stretch, pressure and volume overload (22). Although widely used to support diagnosis and risk prognostication for heart failure (23, 24), elevated NT-proBNP alone does not equate to clinically overt cardiac dysfunction or heart failure; instead, it may signal early subclinical myocardial strain even in the absence of symptomatic cardiac disease. While LVEF remains the gold standard for global systolic assessment, it is often preserved in early diabetic cardiomyopathy, missing subtle dysfunction (25). To overcome this blind spot, we examined both markers: LVEF captures structural pump function, whereas NT-proBNP detects neurohormonal stress that typically precedes overt systolic decline. Together, they cover the full spectrum from subclinical strain to overt dysfunction.

While prior studies reported associations between vitamin D and cardiac biomarkers in general populations (26), evidence in T2DM patients is limited. It remains uncertain whether the association between 25(OH)D and NT-proBNP is independent of diabetes-related metabolic and renal confounders, and whether UACR-reflected systemic microvascular dysfunction acts as a mediator. Few studies have employed DAG-guided causal mediation analysis to formally separate direct and indirect effects. We therefore hypothesized that lower serum 25(OH)D concentrations are associated with greater myocardial strain (reflected by elevated NT-proBNP and reduced LVEF) in T2DM patients, and that this association is partially explained by UACR. To test this hypothesis, we conducted this retrospective study with two predefined objectives: (i) to examine the independent associations of serum 25(OH)D with NT-proBNP and LVEF after adjustment for related confounders; (ii) to quantify the mediating effect of UACR on the relationship between 25(OH)D and myocardial strain using formal causal mediation analysis.

## Subjects and methods

### Study population

Data of T2DM patients who were admitted to the Department of Endocrinology at the People’s Hospital of Jimo, Qingdao between January 2020 and December 2025 were collected. The following criteria were applied for participant selection: (i) individuals diagnosed with type 2 diabetes mellitus and (ii) individuals who underwent serum 25-hydroxyvitamin D detection. Patients with any of the following conditions were excluded: (i) age below 18 years, (ii) history of vitamin D supplementation, (iii) diagnosed with hyperparathyroidism or hypoparathyroidism, (iv) incomplete original data including NT-proBNP, UACR, serum creatinine, left ventricular ejection fraction (LVEF). Finally, 601 participants (210 males, 391 females) were enrolled.

### Measurements

All variables were measured in a single clinical center and data were retrospectively extracted from electronic medical records. We measured height, weight and calculated body mass index (BMI) using the formula: weight (kg) divided by height squared (m^2^). LVEF was calculated by Simpson’s biplane method of disks. Echocardiographic examinations were completed by experienced cardiac sonographers following a standardized institutional protocol using the Philips EPIQ 5 ultrasound system. All participants underwent overnight fasting before venous blood sampling. Diabetes related indicators (fasting blood glucose, C-peptide, glycated hemoglobin), renal function related indicators (SCr, eGFR, UACR), lipid metabolism indicators (triglycerides, low-density lipoprotein cholesterol, high-density lipoprotein cholesterol, total cholesterol), NT-proBNP and 25(OH)D were detected in all subjects. All laboratory tests were completed during the inpatient stay.

Serum 25(OH)D was measured using the Roche cobas e-series analyzer (Roche Diagnostics), with an electrochemiluminescent immunoassay. Routine instrument calibration was performed strictly in accordance with the manufacturer’s instructions. The laboratory reference range for serum 25(OH)D was 0-30 ng/mL. Serum NT-proBNP was determined by magnetic-particle chemiluminescent immunoassay using reagents from Weihai Weigao Biological Technology Co., Ltd. The laboratory reference range was 0-300 pg/mL.

### Diagnostic criteria and cut-off values

In accordance with the Institute of Medicine (IOM) consensus criteria, as operationalized in the Global Consensus on Nutritional Rickets (27, 28), serum 25-hydroxyvitamin D concentrations were categorized as follows: deficiency (<12 ng/mL), insufficiency (12–20 ng/mL), and sufficiency (>20 ng/mL). Based on the 2021 Universal Definition of Heart Failure and the ESC 2021 Heart Failure Guidelines (29, 30), NT-proBNP elevation was defined as a serum level ≥125 pg/mL.

### Covariates identified by DAG

A DAG is a visual representation that illustrates causality between variables, comprising nodes (which represent variables) and directed edges connecting nodes (representing causality between variables) (31). Our research formalized hypothesized causal relationships among variables based on pathophysiological theoretical frameworks, experimental evidence, and clinical domain expertise. Nodes included demographic factors (such as age, sex), anthropometric and clinical factors (including BMI, diabetes duration, HbA1c, hypertension, history of coronary heart disease, eGFR), and core study variables (serum 25(OH)D, UACR, myocardial strain markers including NT-proBNP and LVEF). A DAG was used to identify confounders for multivariable adjustment and to distinguish mediators for subsequent mediation analysis. It revealed the minimal sufficient adjustment sets to evaluate the effects of 25(OH)D on NT-proBNP and LVEF (**Supplementary Figure S1**). The DAG was created according to standard procedures and analyzed with DAGitty 3.0 software.

### Statistical analysis

Statistical analysis was performed using R software version 4.5.3. We used the Kolmogorov-Smirnov test to evaluate whether continuous variables followed a normal distribution. Normally distributed variables were expressed as mean ± standard deviation (SD), and skewed distributed variables were expressed as median and interquartile ranges (IQR). One-way ANOVA or Kruskal-Wallis test was used for comparison of differences between continuous variables among groups. For categorical variables, data are presented as frequency and proportion (%), and group differences were assessed using the chi-square test. To explore the correlations between NT-proBNP and other related indicators, Spearman correlation analysis was performed, with serum 25(OH)D treated as a continuous variable. The effect of 25(OH)D on NT-proBNP was examined using univariate and multivariate logistic regression analyses. Vitamin D status was modeled as a nominal categorical variable with the sufficiency group as the reference, allowing separate estimation of effects for insufficiency and deficiency. As a sensitivity analysis, NT-proBNP was natural-log transformed to reduce right skewness and analyzed as a continuous outcome using multivariable linear regression with the same DAG-identified confounders. Multivariable linear regression was also performed to assess the adjusted association between serum 25(OH)D and LVEF. Mediation analyses were performed using the “mediation” package in R software. Because the outcome model was logistic, the reported effects are on the average marginal probability scale (adjusted risk differences). The significance of the mediation effect was assessed using the bootstrapping technique with 5000 resamples. The significance of the mediation effect was assessed using the bootstrapping technique with 5000 resamples. Results with a two-tailed *P* value below 0.05 were regarded as statistically significant.

### Ethics statement

The study protocol received approval from the People’s Hospital of Jimo, Qingdao (ethical code number: JMPHE0008206002).

## Results

### Population characteristics

A total of 3182 patients with type 2 diabetes mellitus were initially screened. According to predefined exclusion criteria, 2581 patients were excluded, including 32 aged under 18 years, 153 with vitamin D supplementation history, no patients had hyper- or hypoparathyroidism, and 2396 with missing key data (25-hydroxyvitamin D, NT-proBNP, UACR and serum creatinine, etc.). Ultimately, 601 participants were enrolled for analysis. Detailed patient selection is presented in **Figure 1**. Baseline characteristics of included and excluded participants were compared and presented in **Supplementary Table 1**. The median age was 66.6 years old, and 391 (65.1%) of them were female.

**Figure 1:**
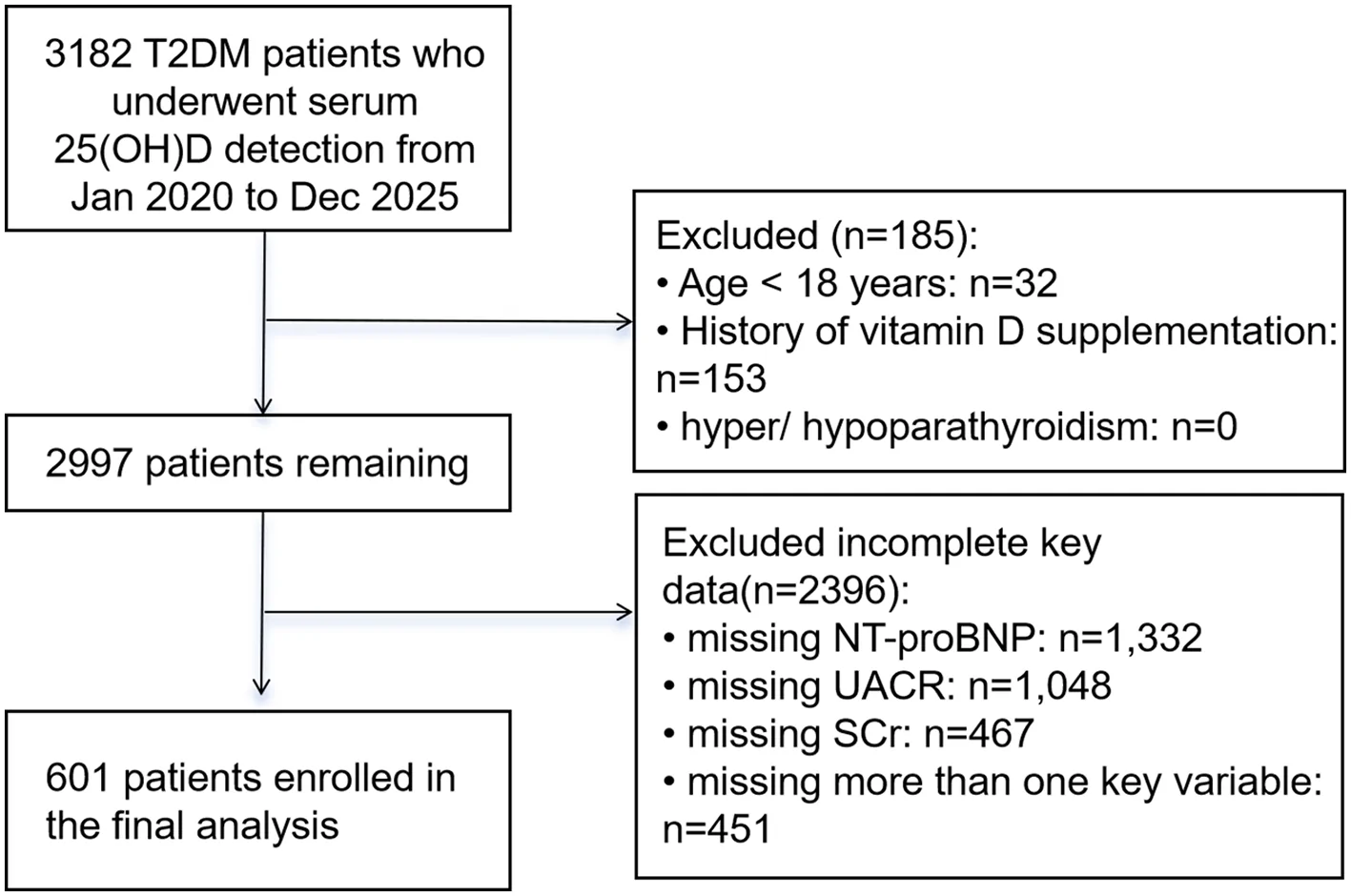
Flowchart of patient selection.

### Baseline characteristics across different groups based on serum 25(OH)D levels

**Table 1** presents the baseline characteristics of patients with different serum 25-hydroxyvitamin D levels. Of all subjects, 143 (24%) presented vitamin D deficiency and 221 (36.8%) presented vitamin D insufficiency. Compared with patients with vitamin D sufficiency, the NT-proBNP levels were markedly increased in patients with vitamin D deficiency (171.80 pg/mL vs. 91.47 pg/mL, *P* < 0.01). The proportion of patients with elevated NT-proBNP (≥125 pg/mL) also differed across groups: 89 (37.6%) in the sufficiency group, 97 (44.1%) in the insufficiency group, and 80 (55.9%) in the deficiency group (*P* = 0.002). SCr and eGFR differed significantly across groups (*P* = 0.008 and *P* = 0.021, respectively). In addition, the UACR levels were significantly higher in the vitamin D deficiency group than those in the sufficiency group (*P* < 0.001). No significant differences were observed in age, BMI, HbA1c, C-peptide, diabetes duration, hypertension, coronary heart disease, TG, HDL-C, LDL-C, TC, ALT, AST, and LVEF among these groups.

**Table 1:** Baseline characteristics of participants in different groups based on serum 25(OH)D levels.

Characteristics	Sufficiency, n=237	Insufficiency, n=221	Deficiency, n=143	*P*
25(OH)D (ng/mL)	25.32 (7.45)	15.34 (4.16)^**^	9.36 (4.34)^**##^	<0.001
Age (years)	66.00 (12)	67.00 (13)	69.00 (13)	0.052
Sex, male, n (%)	108 (45.6)	58 (26.2)	44 (30.8)	<0.001
BMI (kg/m^2^)	24.94 ± 3.81	24.78 ± 3.61	24.73 ± 3.78	0.860
Diabetes duration (years)	11.91 ± 7.33	12.51 ± 7.57	13.50 ± 8.47	0.320
HbA1c (%)	9.20 (3.1)	9.30 (3.0)	9.50 (2.7)	0.527
C-peptide (ng/mL)	1.36 (1.27)	1.34 (1.31)	1.36 (1.21)	0.964
TG (mmol/L)	1.32 (0.88)	1.39 (1.00)	1.36 (1.03)	0.498
HDL-C (mmol/L)	1.18 (0.41)	1.15 (0.37)	1.14 (0.53)	0.992
LDL-C (mmol/L)	2.66 (1.35)	2.72 (1.33)	2.59 (1.32)	0.716
TC (mmol/L)	4.38 (1.56)	4.34 (1.58)	4.24 (1.82)	0.934
AST (U/L)	16.00 (8)	16.00 (7)	14.00 (7)	0.062
ALT (U/L)	16.00 (12)	15.00 (10)	14.00 (10)	0.059
NT-proBNP (pg/mL)	91.47 (145.10)	105.10 (152.88)	171.80 (405.44)^**##^	<0.001
NT-proBNP≥125 pg/mL, n (%)	89 (37.6)	97 (44.1)	80 (55.9)	0.002
LVEF (%)	67.00 (9)	66.00 (7)	65.00 (8)	0.835
SCr (μmol/L)	61.00 (25)	58.00 (20)^*^	61.00 (24)^#^	0.008
eGFR (mL/min/1.73 m²)	92.82 (18.51)	93.28 (17.73)	89.64 (22.92)^#^	0.021
UACR (mg/mmol)	3.0 (6)	3.60 (9)	10.95 (75)^**##^	<0.001
Hypertension, n (%)	166 (70.0)	165 (75.0)	110 (76.9)	0.277
Coronary heart disease, n (%)	130 (54.9)	108 (49.1)	78 (54.5)	0.410
SGLT-2i, n (%)	25 (10.5)	20 (9.1)	15 (10.5)	0.852
GLP-1RA, n (%)	4 (1.7)	2 (0.9)	1 (0.7)	0.620
RAAS inhibitors, n (%)	72 (30.4)	74 (33.6)	48 (33.6)	0.711

Data are presented as median (IQR), mean ± SD or n(%).

25(OH)D, 25-hydroxyvitamin D; HbA1c, glycated hemoglobin; TG, triglyceride; HDL-C, high-density lipoprotein cholesterol; LDL-C, low-density lipoprotein cholesterol; TC, total cholesterol; AST, aspartate aminotransferase; ALT, alanine aminotransferase; NT-proBNP, N-terminal pro-B-type natriuretic peptide; LVEF, Left Ventricular Ejection Fraction; SCr, serum creatinine; eGFR, estimated glomerular filtration rate; UACR, urinary microalbumin-to-creatinine ratio; SGLT-2i, sodium-glucose cotransporter 2 inhibitors; GLP-1RA, Glucagon-like peptide-1 receptor agonists; RAAS inhibitors, renin-angiotensin-aldosterone system inhibitors; SD, standard deviation.

^*^ *P* <0.05, ^**^P <0.01 vs. vitamin D sufficiency; ^#^*P* <0.05, ^##^*P* <0.01 vs. vitamin D insufficiency.

### The correlation between NT-proBNP and clinical variables

As NT-proBNP was shown to be significantly elevated in patients with vitamin D deficiency, we next performed Spearman correlation analysis using NT-proBNP as a dependent variable to identify its risk factors. As shown in **Table 2**, NT-proBNP was negatively correlated with 25(OH)D (r: -0.100, *P* = 0.014), BMI (r: -0.089, *P* = 0.037) and eGFR (r: -0.260, *P* < 0.001), and positively correlated with HbA1c (r: 0.107, *P* = 0.011), SCr (r: 0.260, *P* < 0.001), and UACR (r: 0.123, *P* = 0.004).

**Table 2:** Correlation between NT-proBNP and related clinical variables.

Variables	Correlation coefficient	*P*
age	0.007	0.866
BMI	-0.089	0.037
25(OH)D	-0.100	0.014
LVEF	-0.057	0.237
LDL-C	-0.047	0.260
TG	0.005	0.901
TC	-0.053	0.196
HbA1c	0.107	0.011
C-peptide	0.051	0.220
AST	0.032	0.433
ALT	-0.028	0.502
SCr	0.260	<0.001
eGFR	-0.260	<0.001
UACR	0.123	0.004

NT-proBNP, N-terminal pro-B-type natriuretic peptide; 25(OH)D, 25-hydroxyvitamin D; HbA1c, glycated hemoglobin; LDL-C, low-density lipoprotein cholesterol; TG, triglyceride; TC, total cholesterol; AST, aspartate aminotransferase; ALT, alanine aminotransferase; LVEF, Left Ventricular Ejection Fraction; SCr, serum creatinine; eGFR, estimated glomerular filtration rate; UACR, urinary microalbumin-to-creatinine ratio.

Given that both LVEF and NT-proBNP are key indicators reflecting cardiac function, we also examined the association between LVEF and serum 25(OH)D, as well as other clinical parameters. As shown in **Table 3**, LVEF was not significantly related to serum 25(OH)D, SCr, eGFR, or UACR.

**Table 3:** Correlation between LVEF and related clinical variables.

Variables	Correlation coefficient	*P*
age	-0.071	0.138
BMI	-0.098	0.051
NT-proBNP	-0.057	0.237
25(OH)D	-0.051	0.291
LDL-C	0.027	0.571
TG	-0.030	0.540
HbA1c	0.161	<0.001
C-peptide	-0.019	0.693
AST	-0.035	0.468
ALT	-0.033	0.502
SCr	-0.026	0.589
eGFR	0.029	0.547
UACR	0.011	0.823

NT-proBNP, N-terminal pro-B-type natriuretic peptide; 25(OH)D, 25-hydroxyvitamin D; HbA1c, glycated hemoglobin; LDL-C, low-density lipoprotein cholesterol; TG, triglyceride; TC, total cholesterol; AST, aspartate aminotransferase; ALT, alanine aminotransferase; LVEF, Left Ventricular Ejection Fraction; SCr, serum creatinine; eGFR, estimated glomerular filtration rate; UACR, urinary microalbumin-to-creatinine ratio.

### Logistic regression analysis on correlation between NT-proBNP and vitamin D nutrition status guided by DAG

To further reveal the relationship between NT-proBNP and vitamin D, the patients were stratified into three groups based on the serum 25(OH)D levels. Univariate and multivariate logistic regression analyses were performed, and the results are shown in **Table 4**. In the unadjusted Model 1, compared with patients with vitamin D sufficiency, patients with vitamin D deficiency were associated with higher odds of elevated NT-proBNP (OR: 2.112, 95% CI: 1.385-3.220, *P* < 0.001). After adjusting for prespecified confounders identified by DAG analysis (age, sex, BMI, diabetes duration, hypertension, coronary heart disease, HbA1c, eGFR), patients with vitamin D deficiency still had a significantly higher odds of elevated NT-proBNP (OR: 1.833, 95% CI: 1.115-3.013, *P* = 0.017). In a sensitivity analysis using natural-log transformed NT-proBNP as a continuous outcome, multivariable linear regression showed that 25(OH)D was significantly and inversely associated with ln(NT-proBNP) after adjusting for the same confounders (B = -0.026, 95% CI: -0.038- -0.014, *P* < 0.001), consistent with the primary logistic regression results (**Supplementary Table 2**). After excluding participants with eGFR <60 mL/min/1.73 m² (CKD stage ≥3), the direction and magnitude of associations (OR = 1.764, 95% CI 1.054-2.950, *P* = 0.031) remained broadly consistent with the primary analysis (**Supplementary Table 3**), which demonstrated the robustness of our main findings. After full multivariable adjustment, 25(OH)D showed no statistically significant association with LVEF (B= -1.115, 95% CI: -6.628-3.398, *P* = 0.569; **Supplementary Table 4**).

**Table 4:** Logistic regression analysis on correlation between NT-proBNP and vitamin D nutrition status.

Models	25(OH)D sufficiency	25(OH)D insufficiency		25(OH)D deficiency	
		OR (95%CI)	*P*	OR (95%CI)	*P*
Model 1	1 (Ref)	1.301 (0.895,1.890)	0.168	2.112 (1.385,3.220)	<0.001
Model 2	1 (Ref)	1.391 (0.920,2.103)	0.118	1.985 (1.247,3.160)	0.004
Model 3	1 (Ref)	1.466 (0.944,2.276)	0.088	1.833 (1.115,3.013)	0.017

Model 1: unadjusted model.

Model 2: adjusted for age, sex, BMI, diabetes duration, hypertension and coronary heart disease.

Model 3: adjusted for HbA1c, eGFR on the basis of model 2.

### Mediation effect of UACR on the association between vitamin D deficiency and elevated NT-proBNP

To assess whether the observed association between vitamin D deficiency and elevated NT-proBNP is statistically compatible with an indirect pathway through UACR, an exploratory mediation analysis was conducted. The exposure was vitamin D deficiency versus vitamin D sufficiency; the insufficiency group was excluded because only the deficiency-versus-sufficiency contrast was significantly associated with elevated NT-proBNP in the multivariable logistic regression (**Table 4**). The mediator model was a linear regression of UACR on vitamin D deficiency together with confounders (age, sex, BMI, diabetes duration, hypertension, coronary heart disease, HbA1c, and eGFR). The outcome model was a logistic regression of elevated NT-proBNP on vitamin D deficiency, UACR, and the same confounders. The total effect of vitamin D deficiency on elevated NT-proBNP was 0.277 (95% CI: 0.005-0.539, *P* = 0.038). The direct effect was 0.157 (95% CI: -0.130-0.440, *P* = 0.278), and the indirect effect through UACR was 0.120 (95% CI: 0.009-0.332, *P* = 0.024). Approximately 43.23% of the total effect was statistically compatible with an indirect pathway through UACR (**Figure 2**).

**Figure 2:**
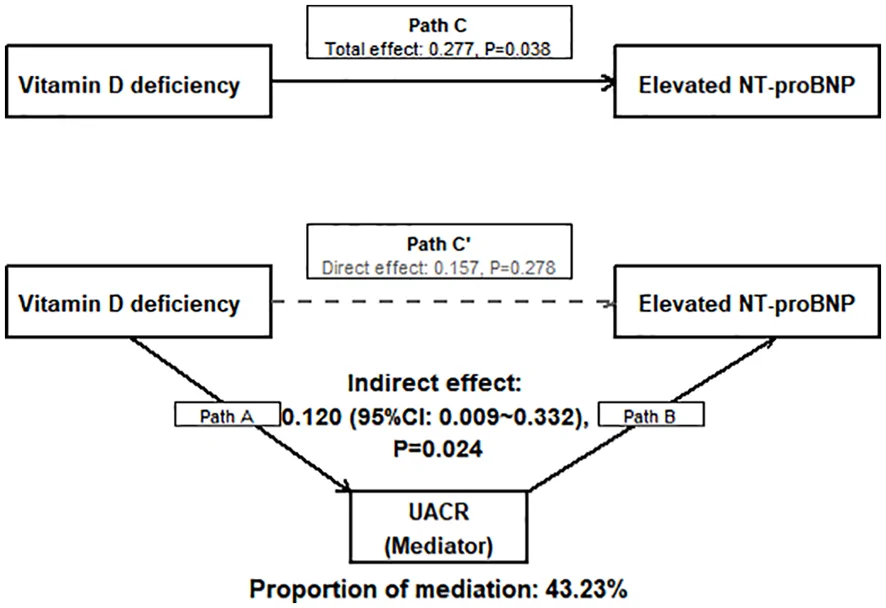
Mediation analysis of UACR in the association between 25(OH)D deficiency and elevated NT-proBNP. Path C denotes the total effect, while path C’ denotes the direct effect. The indirect effect was calculated as the product of path A and path B. The total effect equals the sum of the direct and indirect effects.

## Discussion

Vitamin D, a steroid hormone mainly synthesized in the skin, plays a key role in regulating bone metabolism and calcium-phosphorus balance. It is obtained through skin photoproduction, daily diet and nutritional supplements, with vitamin D3 being the predominant active form synthesized under ultraviolet radiation. The conversion of vitamin D to 25-hydroxyvitamin D occurs in the liver via 25-hydroxylases including CYP2R1 and CYP27A1. The most biologically active form of vitamin D, 1,25-dihydroxyvitamin D, is generated primarily in the renal proximal convoluted tubules through further metabolism of 25(OH)D by 25(OH)D-1α-hydroxylase (CYP27B1) (32, 33). Of all circulating vitamin D metabolites, 25(OH)D is the most abundant and stable (34). Therefore, in this study, the vitamin D nutrition status was classified based on the levels of serum 25(OH)D. Of all the patients, 24% showed vitamin D deficiency and 36.8% presented vitamin D insufficiency, which were quite similar to what was reported by others.

NT-proBNP is a recognized biomarker for myocardial strain and ventricular dysfunction, and its elevation is closely associated with the presence and progression of cardiac dysfunction, even in the early subclinical stage before overt symptoms appear (35, 36). In diabetes, the heart injury and cardiac dysfunction were supposed to be caused by multiple mechanisms. Poor glycemic control induces myocardial injury via the accumulation of advanced glycation end products and oxidative stress, eventually leading to diabetic cardiomyopathy (37). Consistent with this idea, our results showed that NT-proBNP was positively correlated with HbA1c, an indicator for poor glycemic control. Renal dysfunction caused by diabetic nephropathy (DN) is another main contributor to heart injury, particularly to heart failure (38). The primary mechanisms include volume overload due to impaired renal sodium and water excretion, neurohormonal activation, and inflammatory states (39). The elevation of UACR is the early manifestation of DN, which will eventually lead to renal failure, characterized by increased SCr and decreased eGFR. In accordance with the above theory, here we demonstrated that the elevation of NT-proBNP was accompanied by decreased eGFR and increased UACR and SCr. Obesity is a potential risk factor for heart failure, however, our results only showed a weak negative association between NT-proBNP and BMI, which may have no clinical significance.

To examine whether vitamin D deficiency was independently associated with elevated NT-proBNP, we further performed logistic regression analyses. In the univariate logistic regression model, vitamin D deficiency was associated with higher odds (OR 2.11) compared with patients with vitamin D sufficiency. After adjusting for age, sex, BMI, diabetes duration, hypertension, coronary heart disease, HbA1c, and eGFR, the association between vitamin D deficiency and elevated NT-proBNP remained significant. These findings are consistent with previous observations showing an inverse association between 25(OH)D and NT-proBNP in general populations (40, 41). Notably, although the intergroup difference in NT-proBNP reached statistical significance, the absolute difference (80.33 pg/mL between vitamin D deficiency and sufficiency groups) was relatively modest. Combined with the fact that 98.8% of participants had preserved LVEF, this mild elevation in NT-proBNP primarily may reflect early subclinical myocardial strain rather than clinically overt cardiac dysfunction. Based on the pre-specified causal pathway hypothesized via DAG analysis, we speculated that UACR may act as a potential intermediate variable linking vitamin D deficiency and elevated NT-proBNP. To verify this hypothesis, we further conducted formal causal mediation analysis. Results from exploratory statistical mediation analysis revealed that UACR accounted for 43.23% of the total effect of vitamin D deficiency on elevated NT-proBNP, consistent with a mediating role but not definitive proof of biological mediation. It is well recognized that microalbuminuria, as defined by elevated UACR, is not only a marker for diabetic nephropathy, but also a recognized biomarker for systemic microvascular endothelial injury (42, 43). In the general population, microalbuminuria was shown to be an independent risk factor for vascular diseases and heart failure. For example, in the ARIC study of 10–975 individuals, compared with individuals with UACR <5 mg/g, those with UACR of 5–9 mg/g and 10–29 mg/g had adjusted hazard ratios for heart failure of 1.54 and 1.91, respectively, and these associations were independent of coronary artery disease and eGFR. Although not directly measured in this study, one of the hypothesized mechanisms involves the vitamin D receptor (VDR)-eNOS axis. Based on prior experimental work, the activated VDR upregulates eNOS expression and activity via both genomic and non-genomic pathways, including the PI3K/Akt signaling cascade and intracellular calcium-calmodulin pathways, thereby improving NO bioavailability (5, 44, 45). If this pathway operates in our cohort, vitamin D deficiency may compromise these protective mechanisms. In the kidney, glomerular endothelial dysfunction contributes to microalbuminuria. In the heart, microvascular endothelial injury may increase cardiac burden through impaired coronary microvascular perfusion and activation of the renin-angiotensin-aldosterone system (RAAS), and ultimately lead to myocardial strain, reflected by NT-proBNP elevation (35). Direct measurement of eNOS activity, RAAS components, or endothelial function markers would be required to validate these proposed mechanisms.

An important finding of this study is the absence of an association between 25(OH)D and LVEF, which stands in contrast to the significant association observed with NT-proBNP. LVEF reduction usually reflects overt and severe systolic dysfunction; however, it is not a sensitive marker for subclinical heart failure. In our cohort, only 7 patients (1.2%) had overtly reduced LVEF (<45%), and the vast majority exhibited preserved systolic function. Consistently, in the multivariable linear regression adjusting for the same DAG-identified confounders, vitamin D status showed no significant association with LVEF. The lack of correlation between 25(OH)D and LVEF therefore suggests that vitamin D deficiency in T2DM may primarily affect the subclinical stage prior to structural pump dysfunction. This dissociation further supports that elevated NT-proBNP may signal early myocardial strain rather than clinically overt cardiac dysfunction, consistent with the natural progression of diabetic heart disease, in which NT-proBNP elevation generally precedes measurable LVEF decline. Earlier studies showed a close association between vitamin D and LVEF in the general population (46, 47). Such inconsistency with our results may be explained by the fact that our T2DM cohort was largely free of overt heart failure, whereas prior studies may have included patients with more advanced structural disease. We acknowledge that preserved LVEF does not exclude heart failure with preserved ejection fraction (HFpEF), and that elevated NT-proBNP reflects ventricular wall stress rather than established cardiac dysfunction. Accordingly, our cardiac findings should be regarded as exploratory findings, and the relationship between vitamin D status and cardiac structure and function in T2DM patients with overt heart failure warrants dedicated investigation.

Our results identified a negative correlation between serum 25(OH)D and NT-proBNP, and suggested that vitamin D deficiency was observationally associated with subclinical myocardial strain. However, this study has several limitations. First, as a retrospective study, causal relationships cannot be established, and the true temporal ordering among exposure, mediator and outcome variables cannot be determined. Furthermore, unmeasured confounders, such as smoking status, subclinical inflammation, or undiagnosed cardiovascular comorbidities may influence the association between vitamin D status and NT-proBNP, as well as the relationship between mediator UACR and NT-proBNP. Such potential biases could distort the overall association and the estimated indirect effect. The indirect effect should be regarded as exploratory. It shows statistical compatibility with the hypothesized pathway, rather than constituting definitive biological evidence of mediation. Prospective and interventional studies are needed in the future. Second, all participants were recruited from a single medical center. Thus potential selection bias may also exist and the findings may not be generalizable to other populations with different demographic or clinical characteristics. Multicenter studies are needed to validate these results. Third, detailed data on cardiovascular comorbidities and cardiac-related medications were incomplete in electronic medical records, and over-the-counter vitamin D supplementation may not have been fully captured. Fourth, NT-proBNP was used as a surrogate outcome in the present analysis: it reflects ventricular wall stress and does not by itself establish a diagnosis of cardiac dysfunction. Moreover, preserved LVEF does not exclude HFpEF, and the number of patients with overt heart failure was limited. Therefore, the relationship between serum 25(OH)D and LVEF, and with adjudicated heart failure outcomes needs to be further investigated.

## Conclusions

In conclusion, our study demonstrated that patients with T2DM had a high prevalence of vitamin D deficiency/insufficiency. Vitamin D deficiency was associated with elevated NT-proBNP, a marker of myocardial wall stress that may signal early cardiac involvement, whereas no association with LVEF was observed. This association was partially accounted for by UACR in exploratory mediation analyses. Given the retrospective nature of this study, causal inference cannot be drawn. These findings warrant confirmation in prospective and interventional studies.

## Data Availability

The raw data supporting the conclusions of this article will be made available by the authors, without undue reservation.

## Supplementary material

Supplementary Figure 1 Assumed Directed Acyclic Graph (DAG) for the hypothesized relationships among exposure, mediator, and outcome. Green lines denote the hypothesized pathway of interest; pink lines denote potential confounding paths.
