# Supplementary material for: A retrospective study on the associations of serum 25-hydroxyvitamin D with N-terminal pro-B-type natriuretic peptide and left ventricular ejection fraction in patients with type 2 diabetes mellitus
Source: Front Endocrinol (Lausanne). 2026 Oct 28;17:1943266. doi: 10.3389/fendo.2026.1943266 (PMC13752030; doi:10.3389/fendo.2026.1943266)
Supplement: Supplementary file 1 [file Table1.docx]

Supplementary Table 1. Baseline characteristics of included and excluded participants

| characteristics | included, n=601 | excluded, n=2396 | P |
| --- | --- | --- | --- |
| Age (years) | 66.60 (14) | 67.00 (14) | 0.553 |
| Sex, male, n (%) | 210 (34.9) | 886 (37.0) | 0.368 |
| BMI (kg/m^2^) | 24.89±3.70 | 25.24±4.17 | 0.105 |
| Diabetes duration (years) | 14.44±7.79 | 14.27±7.84 | 0.728 |
| HbA1c (%) | 9.40 (3.0) | 9.30 (3.0) | 0.242 |
| TG (mmol/L) | 1.34 (1.27) | 1.32 (1.00) | 0.719 |
| HDL-C (mmol/L) | 1.15 (0.34) | 1.22 (0.39) | <0.001 |
| LDL-C (mmol/L) | 2.66 (1.14) | 2.94 (0.99) | <0.001 |
| TC (mmol/L) | 4.29 (1.45) | 4.65 (1.28) | <0.001 |
| Hypertension, n (%) | 441 (73.4) | 1329(69.84) | 0.799 |
| Coronary heart disease, n (%) | 316 (52.6) | 991 (52.08) | 0.759 |
| SGLT-2i, n (%) | 60 (9.98) | 176 (7.34) | 0.031 |
| GLP-1RA, n (%) | 7 (1.16) | 15 (0.63) | 0.165 |
| RAAS inhibitors, n (%) | 194 (32.3) | 662 (27.6) | 0.023 |

Supplementary Table 2. Multivariable linear regression analysis with log‑transformed NT‑proBNP as the continuous outcome

|  | **B** | **β** | **t** | ***P*** | **95%CI** | **VIF** |
| --- | --- | --- | --- | --- | --- | --- |
| sex | -0.080 | -0.029 | -0.725 | 0.469 | -0.297, 0.137 | 1.062 |
| age | 0.007 | 0.052 | 1.094 | 0.274 | -0.006, 0.021 | 1.495 |
| BMI | -0.081 | -0.228 | -5.592 | <0.001 | -0.109 ,-0.052 | 1.124 |
| 25(OH)D | -0.026 | -0.165 | -4.159 | <0.001 | -0.038, ‑0.014 | 1.060 |
| HbA1c | 0.021 | 0.033 | 0.828 | 0.408 | -0.028, 0.070 | 1.092 |
| eGFR | -0.026 | -0.355 | -7.772 | <0.001 | -0.032, ‑0.019 | 1.403 |
| duration | 0.008 | 0.049 | 1.176 | 0.240 | -0.006, 0.022 | 1.150 |
| hypertension | 0.173 | 0.059 | 1.438 | 0.151 | -0.063, 0.409 | 1.117 |
| Coronary heart disease | 0.144 | 0.054 | 1.342 | 0.180 | -0.067, 0.354 | 1.085 |

Model adjusted for age, sex, BMI, diabetes duration, hypertension, coronary heart disease, HbA1c and eGFR.

Supplementary Table 3. Logistic regression analyses of the association between vitamin D status and elevated NT‑proBNP after excluding patients with eGFR < 60 mL/min/1.73 m².

|  | 25(OH)D sufficiency | 25(OH)D insufficiency | | 25(OH)D deficiency | |
| --- | --- | --- | --- | --- | --- |
|  |  | OR (95%CI) | *P* | OR (95%CI) | *P* |
| Model 1 | 1(Ref) | 1.352(0.911,2.007) | 0.134 | 1.959(1.241,3.092) | 0.004 |
| Model 2 | 1(Ref) | 1.456(0.944,2.245) | 0.090 | 1.899(1.153,3.128) | 0.012 |
| Model 3 | 1(Ref) | 1.471(0.940,2.302) | 0.091 | 1.764(1.054,2.950) | 0.031 |

Note:

Model 1: unadjusted model.

Model 2: adjusted for age, sex,BMI, diabetes duration, hypertension and coronary heart disease.

Model 3: adjusted for HbA1c, eGFR on the basis of model 2.

Supplementary Table 4. Multivariable linear regression analysis for the association between 25(OH)D and LVEF

|  | **B** | **β** | **t** | ***P*** | **95%CI** | **VIF** |
| --- | --- | --- | --- | --- | --- | --- |
| sex | 3.513 | 0.042 | 0.738 | 0.461 | -5.858,12.884 | 1.103 |
| age | -0.463 | -0.101 | -1.520 | 0.129 | -1.063,0.136 | 1.494 |
| BMI | -0.873 | -0.083 | -1.410 | 0.160 | -2.091,0.345 | 1.167 |
| 25(OH)D | -0.115 | -0.025 | -0.441 | 0.659 | -0.628, 0.398 | 1.093 |
| HbA1c | 3.393 | 0.172 | 3.061 | 0.002 | 1.212,5.574 | 1.075 |
| eGFR | -0.077 | -0.036 | -0.564 | 0.573 | -0.348,0.193 | 1.365 |
| duration | -0.575 | -0.106 | -1.765 | 0.079 | -1.215,0.066 | 1.232 |
| hypertension | 9.095 | 0.102 | 1.742 | 0.083 | -1.180,19.370 | 1.160 |
| Coronary heart disease | -1.309 | -0.016 | -0.276 | 0.783 | -10.630,8.013 | 1.164 |

Model adjusted for age, sex, BMI, diabetes duration, hypertension, coronary heart disease, HbA1c and eGFR.
